# Exercise Improves Heart Function after Myocardial Infarction: The Merits of AMPK

**DOI:** 10.1007/s10557-024-07564-2

**Published:** 2024-03-04

**Authors:** Xiaodi Zhang, Yi Zhao, Dafen Guo, Mingxian Luo, Qing Zhang, Li Zhang, Dengshen Zhang

**Affiliations:** 1 Department of Cardiovascular Surgery, Affiliated Hospital of Zunyi Medical University, Zunyi, 563000 Guizhou China; 2 Outpatient Department Office, Affiliated Hospital of Zunyi Medical University, Zunyi, 563000 Guizhou China; 3 Discipline Inspection and Supervision Office of Affiliated Hospital of Zunyi Medical University, Zunyi, 563000 Guizhou China

**Keywords:** Myocardial infarction, AMPK, Exercise, Oxidative stress, Inflammatory response, Mitochondria

## Abstract

**Background:**

AMPK is considered an important protein signaling pathway that has been shown to exert prominent cardioprotective effects on the pathophysiological mechanisms of numerous diseases. Following myocardial infarction, severe impairment of cardiac function occurs, leading to complications such as heart failure and arrhythmia. Therefore, protecting the heart and improving cardiac function are important therapeutic goals after myocardial infarction. Currently, there is substantial ongoing research on exercise-centered rehabilitation training, positioning exercise training as a significant nonpharmacological approach for preventing and treating numerous cardiovascular diseases.

**Objective:**

Previous studies have reported that exercise can activate AMPK phosphorylation and upregulate the AMPK signaling pathway to play a cardioprotective role in coronary artery disease, but the specific mechanism involved remains to be elucidated.

**Conclusion:**

This review discusses the role and mechanism of the exercise-mediated AMPK pathway in improving postinfarction cardiac function through existing studies and describes the mechanism of exercise-induced myocardial repair of AMPK from multiple perspectives to formulate a reasonable and optimal exercise rehabilitation program for the prevention and treatment of myocardial infarction patients in the clinic.

## Introduction

Myocardial infarction (MI), a disease with a high incidence of cardiovascular disease, presents a pressing challenge in the diagnosis and treatment of noncommunicable diseases. The annual increase in incidence and the diagnosis of MI at younger ages underscore the urgency of addressing this issue [1]. Percutaneous coronary intervention (PCI) and coronary artery bypass grafting (CABG), as conventional treatment modalities, can effectively restore coronary artery blood circulation, reestablish reperfusion blood flow, and stabilize postoperative patient mortality at a low level (0.23%) [2, 3]. However, according to the statistics of INTERHEART, the prevalence of depression after acute myocardial infarction (AMI) in China is 21.66%, and the total mortality rate of postinfarction patients is 5.9%, 6.9%, and 7.6% at 30, 60, and 90 days, respectively, threatening the health of the whole population. These findings undoubtedly impose great economic and medical burdens on global public health treatment organizations [4]. Previous studies on the complex pathomechanisms of myocardial infarction have shown that myocardial apoptosis due to acute/chronic ischemia, inflammatory responses, imbalance of oxidative and antioxidant systems, and mitochondrial dysfunction due to internal environmental disturbances exacerbates postinfarction ventricular pathologic remodeling and deterioration of cardiac function [5–8]. Preventing these hazardous pathogenic factors and inhibiting adverse processes are crucial for myocardial infarction salvage and rehabilitation.

## Exercise: Healthy Lifestyle to Disease Treatment

With the rapid development of social pace and people’s lifestyles and dietary changes, the positive effects of exercise training on physical fitness, psychological regulation, weight control, and other positive effects have become a national consciousness, and a variety of “exercise” programs have been pushed into the craze. Various exercise programs have gained popularity, driven by practical observations of rehabilitation patients, clinical data, and experimental studies, including animal in vivo models simulating exercise training. The positive impact of exercise training on human health and well-being has been substantiated through these studies [9]. For instance, exercise training serves as a highly effective preventive measure, reducing the risk of cancer [10, 11]. It is an excellent alternative to primary care medications for older adults with depression [12] and is an excellent means of preventing pregnancy-related disorders and prenatal and postpartum depression during pregnancy [13]. According to data from studies of coronary artery disease, regular cardiac exercise produces new cardiomyocytes at a rate of approximately 7.5% per year, which is 4.6 times greater than that in sedentary populations[14]. Long-term follow-up over > 36 months has shown a significant reduction in cardiovascular mortality as a result of exercise-based cardiac rehabilitation[15]; moreover, according to global surveys of noncommunicable disease data, nearly 9% of premature deaths are attributed to physical inactivity, and exercise-based rehabilitation reduces overall mortality in patients with coronary artery disease by 36–63%[16, 17]. Earlier research demonstrated the regenerative effects of exercise on myocardial cells and tissues, crucial for protecting coronary arteries from ischemic injury. Exercise induces a robust myocardial response in the infarct border zone and stimulates endogenous cardiomyocyte production in ischemia-treated mouse hearts [14]. As early as 1999, for the first time, Yamashitaet’s research team found that exercise reduced the infarct size by nearly 60% [18] in myocardial infarction mice; moreover, this exercise-triggered cardioprotection is activated prior to the onset of the disease, and it continues to be activated through far-reaching recovery from the disease [19, 20]. Mice trained with exercise before ischemic treatment, involving coronary ligation and coronary recanalization after ischemia, exhibited increased myocardial tolerance to ischemia and activation of antioxidant defense factors [21]. Moreover, evidence in medicine explains that exercise excites sympathetic nerves, inhibits parasympathetic nerves, increases catecholamine concentrations, and strengthens myocardial contractility, which slows down the pathological remodeling of the heart muscle after myocardial infarction [22]. An increasing number of patients with coronary artery disease obtain physical protection from exercise training; however, due to the complex pathology of coronary artery disease, exercise training is limited by exercise time, exercise intensity and exercise mode, and the mechanism of myocardial protection during exercise still needs perfecting. Adenylate-activated protein kinase (AMPK), a pivotal regulator of energy metabolism, mediates multiple molecular signaling pathways to protect the body’s energy consumption during myocardial ischemia and hypoxia via the same mechanism of myocardial protection, and exploring the signaling mechanism involved in exercise is important for coronary protection.

### AMPK Structure and Activation

AMPK exists in various eukaryotic cells as a heterotrimer composed of α, β, and γ subunits, with an upstream kinase in the α subunit of the Thr172 residue necessary for AMPK activation [23]. The regulation of its activity is dependent on a change in the ratio of adenosine monophosphate (AMP) and adenosine-5’-diphosphate (ADP) as a way to regulate the phosphorylation of the Thr172α catalytic subunit by the upstream kinase liver kinase B1 (LKB1) and Ca^2+^/CaM-dependent protein kinase (CaMKKβ) [24]. In contrast, the γ subunit contains two pairs of CBS structural domains that bind AMP and ATP and serve as the primary adenosine-5’-triphosphate (ATP) binding domains under normal energy metabolism conditions [25]. AMPK is activated early during energy acquisition and transports membrane vesicles containing glucose transporter type 4 (GLUT4) to the sarcolemmal membrane to accelerate cellular glucose uptake, phosphorylate, and inhibit acetyl coenzyme A carboxylation (ACC) to regulate lipids, and inhibit fatty acid synthesis to regulate energy homeostasis [26, 27]. Hypertrophic adiposity, insulin resistance, and a strong inflammatory response were observed in AMPK knockout mice [28]. When mice were fed β-guanosine propionic acid (GPA), an analog capable of inducing an increase in the AMP/ATP ratio and AMPK activity, the associated increase in mitochondrial enzyme activity led to active mitochondrial biogenesis, whereas there was no effect on AMPK activity or mitochondrial biogenesis-related mitochondrial enzyme activity in transgenic mice with a dominant-negative mutation in AMPK [29]. The α2, β2, γ1, and γ2 isoform forms are most abundantly expressed in regulatory relationships in the heart, especially in ischemic myocardial tissues, where the AMPK γ1 isoform complex accounts for 70% of the cardiac AMPK activity; thus, progressive ventricular hypertrophy due to mutations in the γ2 subunit can be fatal in infants and children when mutations in the γ2 subunit result in progressive ventricular hypertrophy [30, 31]. AMPK serves as an energy sensor, effectively coordinating tissue metabolism to maintain intracellular environmental homeostasis under stress conditions. Its high sensitivity to exercise provides insights into the relationships between the cardioprotective effects of exercise on postinfarction oxidative damage, inflammatory attack and mitochondrial dysfunction and between AMPK signaling molecules. Understanding these relationships offers multiple perspectives on clinical therapeutic targets, drug development, and multifaceted ideas. Although the exact mechanisms underlying myocardial infarction are not fully understood, oxidative stress, inflammation, and mitochondrial dysfunction are involved in this process.

### AMPK in Exercise Contraction

In earlier studies, AMPK has been proposed to mimic the effects of exercise [32], which means that the pharmacological effects of AMPK in disease can be realized through exercise activation. This approach aims to transform exercise from a “healthy lifestyle” to a “nonpharmacological therapeutic tool”. Skeletal muscle exercise induces various effects, marked by a significant increase in muscle energy turnover and alterations in nucleotide status. During exercise, ADP, a product of ATP hydrolysis, is rapidly converted to AMP by the adenylate kinase reaction, and the intracellular AMP/ATP ratio increases. This energetic stress causes AMP to bind to the CBS structural domain of the γ-subunit of AMPK to activate AMPK, promoting the phosphorylation of AMPK at the α-subunit of Thr172, which is capable of linearly increasing its activity depending on the intensity of the exercise by more than 100-fold, and this change is accompanied by a change in nucleotide status [33–36]. Western immunoblotting experiments revealed the activation of key signaling pathways, including the AMPK (Thr172) pathway, and the phosphorylation of the AMPK substrate acetyl coenzyme A carboxylic acid (ACC; S79) site in muscle after both in situ contraction stimulated by the sciatic nerve and treadmill exercise [37]. However, the different isomeric forms show different endpoints during exercise contractions depending on differences in exercise intensity and duration, with an increase in AMPKα2 activity observed at 70% VO_2max_ exercise intensity for only 20 min, whereas AMPKα1 does not show significant changes in this exercise pattern and is activated only by intense exercise or strong physical body external electrical stimulation [38, 39].

In cardiac metabolism, glucose uptake, fatty acid uptake, and protein synthesis are important for the repair of damaged cardiac tissues. In ischemic and hypoxic myocardium, activated AMPK drives the translocation of GLUT-4 to increase glucose uptake in myocardial tissues, but, interestingly, the specific knockout of AMPKβ1β2 in mouse muscle tissues did not reverse the accelerated degradation of ATP and the concomitant decrease in glucose uptake in skeletal muscle during exercise, even with exercise, which seems to demonstrate that AMPK plays a central role in the regulation of homeostasis [40–42]. In contrast, when AMPKα was absent or severely inactivated, the total phospholipoprotein content of mice in the AMPKα-deficient group was still 20% higher than that of mice in the wild group, although it could be reduced by exercise. Meanwhile, the ratio of phosphorylated AMPK Thr172: total AMPKα in AMPK-deficient mice was approximately 65% lower compared with that in wild mice, and the cardiac endoplasmic reticulum Ca^2+^-ATPase 2a (SERCA2a) was reduced by approximately 37%. That is, exercise can trigger cardioprotective mechanisms in the presence of cardiac dysfunction, but only with the involvement of AMPK phosphorylation [43].

Different isoforms activated by the sensitivity of AMPK to exercise and the variety of exercise forms play different functional roles and are important for coronary and myocardial protection.

### Oxidative Stress in AMPK

In CVD, the relationship between AMPK and oxidative stress involves mutual regulation and constraints. Oxidative stress can modulate the activity of AMPK, and, reciprocally, activated AMPK plays a crucial role in upregulating the expression of endogenous antioxidant genes, serving as a defense mechanism against oxidative damage primed by reactive oxygen species (ROS). Simultaneously, activated AMPK stimulates peroxisome proliferator-activated receptor-gamma coactivator-1α (PGC-1α), contributing to mitochondrial biogenesis [30, 44]. Following a myocardial infarction, the myocardium undergoes ischemic and hypoxic stress, leading to the rupture of the mitochondrial respiratory chain. This process results in an accumulation of metabolites such as peroxides, superoxides, and hydroxyl radicals, collectively referred to as ROS, and a depletion of antioxidants [22]. This imbalance in oxidative and antioxidative processes triggers oxidative stress, causing damage to cellular membrane lipids and mitochondrial DNA. This, in turn, disrupts the integrity of cardiomyocytes, increases cellular permeability, and exacerbates cellular damage and apoptosis [25]. Exogenous AMPK activators significantly increase the expression of antioxidant factors, including catalase (CAT), superoxide dismutase (SOD) 1, SOD2, and uncoupling protein 2 (UPC2), and inhibit ROS production during hypoxia and reoxygenation in H9c2 cells in vivo [45, 46]. Specifically, under stressor stimulation, AMPK is able to respond sensitively to oxidative damage caused by the myocardium by blocking the production of superoxide from mitochondria or nicotinamide adenylate dinucleotide phosphate oxidase (Nox) while directly driving the activation of downstream antioxidant genes and promoting the migration and aggregation of nuclear factor erythroid-2-related factor 2 (NRF2) into the nucleus [47–49]. NRF2 is considered a nonnegligible major regulator of antioxidant defense that prevents cells from oxidative damage [50] and may become an important therapeutic strategy to prevent oxidative stress [51].

### AMPK and Oxidative Stress During Exercise

Resveratrol, as an AMPK activator, induces AMPK activation by significantly reducing the level of NADPH oxidase, inhibiting ROS production, and increasing myocardial antioxidant enzyme activity [52]. This effect contributes significantly to the amelioration of myocardial injury. Interestingly, exercise training demonstrates similar myocardial protective effects as resveratrol, acting as an effective agonist of AMPK [53]. Swimming exercise activates SIRT1/AMPK/NRF2-mediated lipid metabolism and prevents the secretion of substrates involved in oxidative stress [54]. Angiotensin II (AngII) in the vasculature also regulates ROS production because an increase in AngII in the infarcted myocardium is accompanied by an increase in ROS content and NADPHase activity; however, after exercise training intervention, with the activation of AMPK, AngII expression decreases, and NADPHase activity is subsequently inhibited, preventing ROS biosynthesis [54]. This view is consistent with the findings of Geolotto et al., who concluded that the inhibition of NADPH oxidase activity reduces ROS production and that this effect is significantly enhanced by AMPK activators and significantly diminished by intervention with AMPK inhibitors [55]. In summary, exercise can function as an exogenous AMPK activator, exerting cardioprotective effects by enhancing antioxidative defense against myocardial infarction. This nonpharmacological research strategy proves highly effective, and its mechanism is closely associated with the AMPK signaling pathway.

### Inflammatory Responses in AMPK

Systemic and local inflammation after myocardial infarction is widely accepted to accompany the onset to the end of MI. Coronary hypoxia disrupts vascular endothelial cell integrity and barrier function, leading to the infiltration of myocardial tissues with C-reactive protein aggregates in peri-infarct zones. Proinflammatory factors, such as serum tumor necrosis factor-alpha (TNF-α) and interleukin-6 (IL-6) are released [56], initiating an inflammatory cascade characterized by the recruitment of inflammatory cells, chemokines, and cell adhesion factors into the bloodstream [28, 57]. In this process, MI-triggered activation of nuclear factor-kappaB (NF-κB) and its subsequent nuclear translocation are considered to be the central effectors of inflammatory signaling, and this triggered inflammation promotes myocardial injury, repair, and scarring, leading to myocardial dysfunction and remodeling [58]. However, interestingly, the induced inflammatory response during the infarction process shows contradictory results, which in general means that inflammation during myocardial infarction is a double-edged sword for cardiac repair [57]. The inflammatory response occurs during myocardial ischemia and reperfusion and in the early phase of acute myocardial ischemia. Lipid metabolism restricts the inflammatory threat of proinflammatory cells to the myocardial myocardium by converting leukotriene B4 (LTB4) synthesis into lipid mediators [59, 60], and exogenous TNF-α stimulation has been suggested to increase rapamycin-induced myocardial autophagy and effectively prevent cardiomyocyte apoptosis[54]. However, in the later phase, persistent stimulation of NF-κB exacerbates reactive oxygen intermediates (ROIs) and nitric oxide (NO) production, and the myocardium is subjected to intense inflammatory attack with simultaneous activation of oxidative stress; moreover, the inflammatory response transforms itself into a key factor in accelerating myocardial dysfunction [60]. Liraglutide can treat myocardial inflammatory responses caused by metabolic disorders and mitochondrial dysfunction due to its activation of AMPK protein activity, which inhibits the inflammatory expression of interleukin 1β in cardiomyocytes [61], leveraging the classical anti-inflammatory properties of AMPK [54, 62].

### AMPK and Inflammation in Exercise

The anti-inflammatory effects of exercise were revealed as early as the 1980s [63]. According to previous studies, exercise-induced AMPK signaling significantly ameliorates inflammation induced by high-fat factors [64]. Both aerobic and booster training promote the release of anti-inflammatory cytokines (IL-10), inhibit TNF-α secretion, and ameliorate cardiac fibrosis to reduce pathological myocardial remodeling [65, 66]. Although there is little direct evidence of the anti-inflammatory effects of exercise in relation to the AMPK mechanism, the evidence is summarized that exercise significantly promotes the release of the anti-inflammatory factor IL-10 and the interleukin IL-1 receptor antagonist and inhibits the secretion of TNF-α while blocking the inflammatory response of the Toll-like receptor pathway.

### Mitochondrial Dysfunction in AMPK

Mitochondria play a crucial role in providing ATP energy, serving as the foundation for various tissues and organs to fulfill their metabolic functions [67]. Mitochondria are regarded as networked regulatory systems by virtue of their extraordinary morphological and structural plasticity. Regardless of physiological renewal and development or defense resistance under stressor stimuli, mitochondria achieve self-balancing and quality control through biogenesis, fission, fusion, and self-phagocytosis in response to cellular and environmental demands. In the heart, cardiomyocytes boast a robust mitochondrial network, constituting up to 30% of the heart volume. Oxidative phosphorylation within these mitochondria generates the energy necessary to sustain the high-density function of the heart. However, after myocardial infarction, ATP synthesis is impeded by continuous hypoxia and disruption of nutrient supply to cardiomyocyte tissues [68, 69]. At the same time, aerobic metabolism is impaired due to hypoxia in cardiomyocytes, and the mitochondrial environment is disrupted, leading to an imbalance in the acid‒base balance. This imbalance results in changes to acid‒base equilibrium, alterations in intramembrane permeability, Ca^2+^ entry into mitochondria, damage to the electron transport chain, and an excess of mitochondrial ROS and calcium overload. These factors collectively suppress normal biological functions [22, 70]. Interestingly, PGC-1α, a key regulator of mitochondrial biogenesis and a downstream AMPK protein pathway, can directly phosphorylate the PGC-1α protein at threonine 177 and serine 538, stimulating mitochondrial biogenesis and contributing to energy production [71–73]. Specific knockdown of the AMPKα subunit induces mitochondrial biogenesis as well as functional deficits [74]. An increasing number of studies have revealed the importance of AMPK in the regulation of mitochondrial homeostasis. AMPK is important for the regulation of mitochondrial homeostasis [75, 76].

### AMPK and Mitochondrial Dysfunction in Exercise (Fig. 1)

**Fig. 1:**
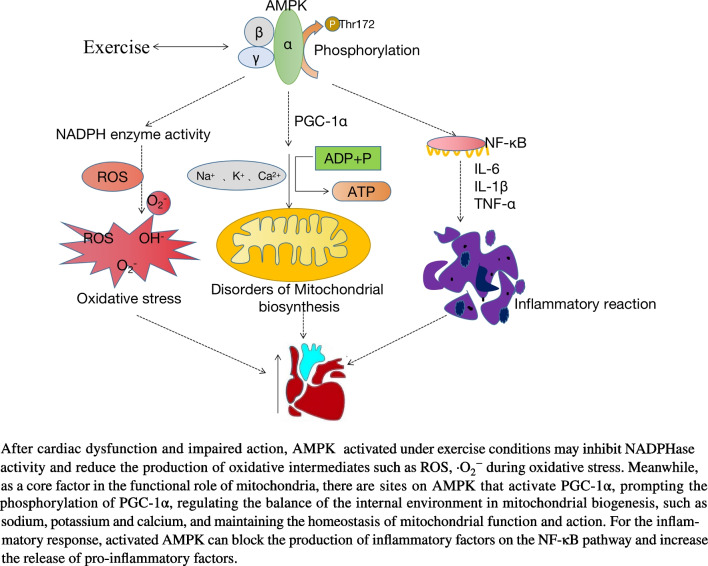
Mechanism of exercise and AMPK after myocardial

The remarkable structural plasticity of mitochondria empowers them to modify their network structure through exercise training, resulting in a substantial increase of approximately 40–50% in total mitochondrial proteins. This alteration significantly enhances mitochondrial antioxidant capacity, attributed to Mn- and Cu-, Zn-superoxide dismutase and catalase, while also boosting overall mitochondrial function [73, 77]. The exogenous AMPK agonist AICAR significantly induces mitochondrial biogenesis in skeletal muscle after simulated exercise intervention [60]. The master transcriptional regulator PGC-1α, which plays a key bridging role, was identified [78]. Additionally, the increased PGC-1α and vascular endothelial growth factor (VEGF) action that accompanies exercise promotes the level of the core factor of neovascularization, HIF-1α mRNA, and this neovascularization also benefits from the role of AMPK activation [79, 80]. Animal studies have shown that significant activation of AMPK after acute exercise or exercise training is accompanied by an increase in ULK1 phosphorylation, and that activated AMPK is able to translocate ULK1 to mitochondria via phosphorylation at the serine 555 site [81, 82]. While specific deletion of AMPK in muscle tissue leads to mitochondrial damage, consistent with the mitochondrial dysfunction exhibited by ULK1-loss mice, it can be argued that exercise drives mitochondrial mitophagy, but this requires the involvement of the AMPK-ULK1 regulatory axis, and that the resulting autophagy is highly dependent on the intensity of the exercise and the AMPK activity [83].

In a mouse model of high-fat diet-induced diabetic cardiomyopathy, oxidative phosphorylation and the increase in the membrane potential were significantly enhanced through the AMPK/PGC-1α signaling pathway, ROS production and oxygen consumption were reduced, and myocardial damage caused by mitochondrial dysfunction and lipid metabolism disorders was ameliorated [84]. Similarly, during exercise, exercise activates the AMPK/PGC-1α signaling pathway, which promotes ATP production and increases mitochondrial biosynthesis [85], which implies that none of the genes involved in mitochondrial metabolism seem to be indispensable for the regulation of the PGC1 family through interactions with estrogen-associated receptors (ERRs) [86]. In addition, through fission and fusion associated with mitochondria after exercise, the minute expression of electron transport chain proteins of the tricarboxylic acid cycle machine is increased [2], which is similar to the role of AMPK and will lead to the exploration of new mechanisms linking exercise and AMPK.

## Future Prospects

As previously mentioned, ischemia/reperfusion injury stemming from sustained ischemia/hypoxia and the subsequent restoration of reperfusion after infarction are significant contributors to the exacerbation of the infarction process. These processes involve oxidative stress, inflammation, and disrupted mitochondrial energy metabolism, which, if not addressed promptly, contribute to undesirable pathways. A large number of experiments have proven that AMPK signaling has a beneficial effect on reversing these undesirable pathways, and studies on the activation of the AMPK signaling pathway through exercise have also been reported. Research has also shown that this approach is undoubtedly beneficial to patients, regardless of the economic pressure on patients and recovery after intervention. Moreover, from the perspective of clinical care, in-depth studies of exercise-mediated rehabilitation mechanisms also provide nursing staff with a clearer theoretical support for patients to carry out disease and exercise rehabilitation from empty “empiricism” to solid knowledge of rehabilitation. The change from empty “empirical” to solid “theoretical perception” of the knowledge of disease and exercise rehabilitation for patients is clinically feasible and beneficial to patients. However, the specific protein pathways and targets through which exercise-mediated AMPK improves postinfarction cardiac function have yet to be studied in depth. Additionally, the safety of exercise training has not been definitively guaranteed in practical terms. Therefore, a thorough assessment of the operation and preparation of emergency plans for unforeseen safety incidents should precede exercise rehabilitation. Finally, this review aimed to illustrate the significance of nonpharmacological forms of exercise training in mediating AMPK phosphorylation on cardiac function after myocardial infarction, to further promote the value of cardiac rehabilitation and clinical care compliance and to improve the prognosis and quality of life of patients with myocardial infarction.

## Data Availability

All the information in this article is open and transparent.
